# The Role of Zeolite Structure in Its β-cyclodextrin Modification and Tetracycline Adsorption from Aqueous Solution: Characteristics and Sorption Mechanism

**DOI:** 10.3390/ma15186317

**Published:** 2022-09-12

**Authors:** Lidia Bandura, Monika Białoszewska, Tiina Leiviskä, Małgorzata Franus

**Affiliations:** 1 Department of Construction Materials Engineering and Geoengineering, Faculty of Civil Engineering and Architecture, Lublin University of Technology, Nadbystrzycka 40, 20-618 Lublin, Poland; 2 Chemical Process Engineering, University of Oulu, P.O. Box 4300, FIN-90014 Oulu, Finland; 3 Department of Construction, Faculty of Civil Engineering and Architecture, Lublin University of Technology, Nadbystrzycka 40, 20-618 Lublin, Poland

**Keywords:** β-cyclodextrin, zeolite modification, antibiotics, adsorption, tetracycline

## Abstract

Modification of zeolites with organic compounds is of increasing interest due to their significant potential in removing emerging pollutants from water. In this work, zeolites from fly ash with three different structure types, NaX (faujasite), NaA (Linde A) and NaP1 (gismondine), were modified with β-cyclodextrin (β-CD), and their adsorption efficacy towards tetracycline (TC) antibiotic in aqueous solutions have been studied. To assess the effect of modification on the zeolites, they were subjected to chemical, mineralogical and surface analyses using X-ray diffraction (XRD), thermogravimetry (TG), scanning electron microscope (SEM), N2 adsorption/desorption isotherm, Fourier-transform infrared spectroscopy (FTIR), and X-ray photoelectron spectroscopy (XPS). The maximum adsorption capacity for NaX-CD, NaA-CD and NaP1-CD was around 48, 60, and 38 mg/g, respectively. The fastest adsorption rate was observed for NaP1-CD, which achieved adsorption equilibria after 200 min, while for NaX-CD and NaA-CD it was established after around 24 h. The kinetic data were best described by the Elovich model, followed by pseudo-second order, while the Sips and Redlich–Peterson models were the most suitable to describe the adsorption isotherms. Based on the adsorption data as well as FTIR and XPS results, TC adsorption efficacy is strongly related to the amount of CD attached to the mineral, and hydrogen bonding formation probably plays the major role between CDs and adsorbate.

## 1. Introduction

Antibiotics are widely used throughout the world in human and veterinary medicine to treat diseases. However, their excessive consumption can be observed nowadays, especially in countries where antibiotics can be easily purchased without a prescription, as well as in mass livestock farming [1]. Mass consumption of antibiotics, their improper use [2], as well as overuse in animal farms contributes to environmental pollution by these substances as they are excreted by living organisms in their unchanged or metabolized form. Additionally, the pharmaceutical and medical industries as well as the antibiotics disposal cause these compounds to enter wastewater from which they end up in groundwater, drinking water, and soils [3]. Moreover, antibiotics have the ability to accumulate in the environment, and conventional wastewater treatment processes do not remove antibiotics well [2,4,5]. These hazardous pollutants contribute to the growth of drug-resistant strains of bacteria, which is now the subject of increasing concern and requires reasonably planned actions on the global scale [2,6]. Tetracycline (TC) is among the wide-spectrum antibiotics and it is very commonly used in many therapies. TC is hard to remove in conventional biological treatment processes [7]. Hence, it is of utmost importance to search for effective methods to remove this type of emerging contaminants from water bodies.

Among water treatment technologies several methods can be used for organic pollutants such as advanced oxidation [8], membrane-assisted processes, biodegradation, ozonation and adsorption [9]. Adsorptive methods are considered to be efficient, affordable and low-energy consuming, as well as easy to perform. Carbon materials, such as activated carbon AC, are the most popular and effective adsorbents employed for the removal of various pollutants in waters. However, AC has some limitations and disadvantages such as high costs, non-selectivity and difficulties in regeneration [10]. Thus, cheaper alternatives such as biomass-derived adsorbent [11], mineral and organo-mineral adsorbents [12], and waste-derived porous materials [13,14] have been developed and studied in terms of antibiotics removal.

Zeolites, due to porous structure and other unique properties, are applied in environmental engineering [15,16,17,18], civil engineering [19,20,21], and agriculture [22]. They are frequently used as efficient adsorbents for various types of environmental pollutants, especially in water treatment technologies for the removal of heavy metals [23], organics [24], radionuclides [25], or ammonium ions [26]. Zeolites are hydrated skeletal aluminosilicates of alkali elements, alkaline earth metals, or other cations, composed of tetrahedrons connected by their corners via shared oxygen atoms. They are distinguished by a great variety of mineral structures, forming systematic networks of cavities and chambers of different sizes (usually around 3–30 angstroms in their diameter). Metal cations are present in these channels (mostly Na^+^, K^+^, Ca^2+^), compensating for the negative charge resulting from the crystal lattice structure of the zeolites. Zeolite channels also carry water molecules called “zeolitic water”. Natural and synthetic zeolites can be distinguished. In the group of natural zeolites, around 100 of different mineral structures are defined; however, just a few of them occur in deposits that are accessible for mining and processing (clinoptilolite, philipsite, chabazite, mordenite). In the case of the synthetic ones, more than 200 framework types have been obtained so far. Generally, they can be obtained from chemical reagents (sodium silicate and sodium aluminate) but also from industrial waste such as fly ash [27], perlite [28], kaolin [29], and obsidian [30]. It is precisely their ability to be derived from industrial waste that makes these adsorbents attractive and relevant to cleaner production and sustainability goals.

Each zeolitic structure has a three-letter code provided by The International Zeolite Association (IZA) Structure Commission (e.g., SOD for sodalite, LTA for Linde A, GIS for gismondine, FAU for faujasite). This code is a part of the official International Union of Pure and Applied Chemistry (IUPAC) nomenclature for microporous materials [31].

The structures of synthetic zeolites, in addition to the above code, are assigned proper names, which have been widely adopted in scientific nomenclature, and among the most commonly utilized synthetic ones are the structures of Na-X, Na-Y, Na-A, Na-P1, ZSM-5 [32]. In order to enhance their adsorption ability towards organic and/or anionic compounds occurring in waters, zeolites have been subjected to modifications using organic compounds such as, in most cases, quaternary ammonium salts [33], chitosan [34] or inorganic elements such as lanthanium [35]. More rarely, examples of modifications using cyclodextrins (CDs) can be found in the literature [36,37,38,39]. CDs belong to the oligosaccharide compounds of a cone shape with hydrophobic interior and hydrophilic outside surface. CDs are non-toxic and can form host–guest complexes with many organic compounds, and therefore, they are considered as effective surface modifiers for mineral adsorbents [40].

According to the current state of the art, it is known that zeolites and other mineral materials can be easily modified by CD in a view to use them for the broad range of organic pollutants adsorption from water systems. However, the influence of the structure type and textural properties of minerals from the same group on the CDs modification efficacy have not been reported before. Additionally, the bearing of the modification effect (in terms of quantity and quality) on the adsorption efficiency of tetracycline on fly ash zeolites modified with CD has not yet been studied. In this work, fly ash derived zeolites of three different structure types, NaX (FAU), NaA (LTA), NaP1 (GIS), have been modified by β-CD according to the procedure reported previously [36]. In order to assess the effect of modification, raw zeolites and the obtained hybrid forms (NaX-CD, NaA-CD and NaP1-CD) have been characterized in terms of mineral and chemical composition as well as textural properties and morphology. TC adsorption tests were then performed, including the effect of adsorbent dosage, contact time and initial concentration. Several isotherm and kinetic models have been applied to fit the experimental sorption data to evaluate the adsorption mechanisms. In addition, adsorbents after TC adsorption have been characterized using FTIR and XPS with a view to reveal changes in the materials character after adsorption process. This work is essential for the development of mineral adsorbent functionalization techniques, and for the evaluation of their use as alternative and sustainable adsorbents for the removal of organic micropollutants from aquatic environments.

## 2. Materials and Methods

### 2.1. Materials

Fly ash was obtained from Jaworzno III power plant (Poland). The chemical composition is presented in Table 1. Sodium hydroxide, methanol, ethanol and acetone were purchased from P.P.H. “Stanlab”, Lublin, Poland. Aluminum metal chips were purchased from “Pol-Aura”, Poland. β-cyclodextrin (β-CD), (3-Glycidyloxypropyl)trimethoxysilane (GLYMO), *N,N*-Dimethylformamide (DMF), sodium (Na), tetracycline hydrochloride (TC) were purchased from Sigma-Aldrich Company.

Zeolites of NaX, NaA and NaP1 structures were produced using a technological line for zeolite synthesis from fly ash reported by Wdowin et al. [41]. This equipment consists of four process blocks: the reactor loading station, main reactor, reaction product separation module, and final material processing unit. Briefly, weighted amounts of fly ash and granulated sodium hydroxide are fed into the main reactor, to which an adjusted amount of water is then added. Then, the transformation of fly ash into zeolite material is carried out, which is known as the hydrothermal conversion. The conceptual scheme of this process can be presented as follows:

$$fly\;ash+x\;\left[mol\cdot \;dm^{-3}\right]\;NaOH\begin{array}{c} time\\ \to \\ temperature \end{array}zeolite+residuum$$

Using variable parameters of the synthesis, such as substrate concentration, temperature of the conversion process, and reaction time, it is possible to obtain zeolites with different types of structures. The conditions applied in the synthesis process are summarized in Table 2.

NaX and NaA (faujasite (FAU) and Linde A (LTA) types, respectively) are composed of sodalite unit (SOD) formed from 24 T-atoms (so-called β-chambers, composed of six 4-rings, four 6-rings, three 6-2 units or four 1-4-1 units). In the case of NaX, the β-chambers are connected to each other by a double 6-membered ring D6R. Between the β-chambers, a large “super-cage” is formed with an inner diameter of about 13 Å, surrounded by four 12-member rings of 7.4 Å diameter. The NaA (LTA) is formed by sodalite units connected by a double 4-membered ring D4R. In cubic LTA, the 8-ring channels are arranged parallel to [100] direction forming an α-cavity with dimension of 4.1 × 4.1 Å. Na-P1 belongs to the gismondite group (GIS) in which two 4-membered rings form an 8-membered channel with dimensions of 3.1 × 4.5 Å and 2.8 × 4.8 Å along the [100] and [010] directions, respectively.

In order to modify zeolites with CD, 3 g of β-CD was thoroughly dissolved in 100 mL of DMF using a magnetic stirrer. Next, 0.3 g of sodium was added and stirred for 2 h at room temperature in order to obtain β-CD-Na salt. The solution was filtered from the unreacted Na, and 2 mL of the linking agent (GLYMO) was added. The mixture was stirred, at 85–90 °C, for 5 h. Next, 3 g of previously dried zeolite material was added and the mixture was stirred overnight, at 110–120 °C. Obtained material was filtered, washed sequentially by DMF, deionized water, methanol, and acetone, and dried at 105 °C for 24 h.

### 2.2. Characterization of the Materials

Mineral composition was determined using X-ray diffraction XRD (Panalytical, Almelo, The Netherlands). The analysis was conducted with the powder method using X-ray diffractometer Panalytical X’pert PROMPD with a goniometer PW 3050/60 in the angle range 5–65 2θ. A copper lamp Cu (CuKα = 0.154178 nm) was applied as the source of X-radiation. Diffraction data processing was performed by X’ Pert High Score software. Mineral phases identification was based on PDF-2 release 2010 database, formalized by JCPDS/ICDD.

TG analysis was performed using the thermal analyzer STA 449 F1 Jupiter (Netzsch, Germany). The TG curves were recorded over the 30–950 °C range, at a heating rate of 10 °C/min, in the synthetic air atmosphere (50 mL min^−1^). The samples (~7 mg) were placed in a alumina crucible. An empty Al_2_O_3_ crucible was used as a reference.

The morphology of the materials was investigated using Scanning Electron Microscope (SEM) Quanta 250 FEG by FEI (Hilsboro, OR, USA).

Textural parameters were determined by the nitrogen adsorption/desorption measurements, at −196 °C, using a Micromeritics ASAP 2020 instrument (Norcross, GA, USA). The specific surface areas, *S_BET_* [m^2^/g], were calculated using the standard Brunauer−Emmett−Teller (BET) equation. The total pore volumes, *V_tot_* (cm^3^/g), were calculated from a single point adsorption at *p*/*p_0_* = 0.98.

The electrophoretic mobility (Ue) of the examined zeolite suspensions was measured in the pH range 3–11 (containing 0.07 g of the solid for 100 cm^3^ of the NaCl solution) using Zetasizer Nano ZS (Malvern Instruments). Electrophoretic mobility of solid particles dispersed in the liquid medium was measured using the dip cell (five repetitions of measurements for each sample). The zeta potential (ζ) was calculated with the special computer program using the Henry equation. The isoelectric point (IEP) of zeolites was also determined.

The raw zeolites and their modified form before and after TC adsorption were characterized by infrared spectroscopy (FTIR) and X-ray photoelectron spectroscopy (XPS). The FTIR spectra were measured a using the DRIFT technique. The powder samples were dried at 105 °C, overnight, before measurements, and were then ground in a mortar with KBr (3% wt. sample/KBr). FT-IR/DRS spectra were recorded on a Nicolet 380 spectrophotometer (Thermo Scientific, Waltham, Massachusetts, USA). The spectra were recorded in the range of 4000–400 cm^−^^1^, at room temperature, with a resolution of 4 cm^−^^1^ and a mirror velocity of 2.5 kHz. The interferograms consisted of 1024 scans, which guaranteed a good signal/noise ratio. The spectra were normalized by comparing the obtained spectrum to the background spectrum (spectrum of dried and triturated KBr). No smoothing functions were used. The X-ray photoelectron spectroscopy XPS was conducted using a Thermo Fischer Scientific ESCALAB 250xi with a monochromatic Al Kα source (1486.6 eV). Before the measurements, powdered samples were ground and dried overnight, at 105 °C for 24 h, The XPS measurements were conducted by placing the samples on a gold coated sample holder. Tha base pressure of the XPS analysis chamber was 5 × 10^−9^ mbar. The charge correction was performed by setting the binding energy of adventitious carbon to 284.8 eV. C for 24 h.

### 2.3. Batch Adsorption Tests

To perform batch adsorption experiments, the TC stock solution of 200 mg/L were prepared in 1 L volumetric flask. The effect of the adsorbent dose (0.5; 1; 2; 3; 4; 5 g/L), contact time (1 min–72 h), and initial concentration of adsorbates (5–200 mg/L) on the adsorption process were studied. Each experimental set was performed at room temperature, without pH adjustment, in a 50 mL conical flasks, and the volume of adsorbate solutions was 25 mL. The flasks were agitated at a constant speed of 120 rpm for an appropriate time intervals. After the adsorption process, the adsorbent was separated by centrifugation (2500 rpm for 10 min). Simultaneously, blank samples with adsorbents in 25 mL of distilled water were provided to exclude the influence of the material on absorbance measurements. Control solution samples without the adsorbent were prepared to exclude the influence of volatilization on the absorbance values. All experiments were performed in duplicate.

The concentration of TC solutions before and after adsorption tests was measured using UV–Vis spectrophotometer (Helios gamma, Thermo Scientific) at the wavelength of 357 nm (Table S1) [3,13].

The percentage of the TC removal *R*(%), and the equilibrium adsorption capacity *q_e_* (mg/g) were calculated using the following equations, respectively:

(1) $$R\left(\%\right)=\frac{100\left(C_{0}-C_{e}\right)}{C_{0}}$$

(2) $$q_{e}=\frac{\left(C_{0}-C_{e}\right)V}{m}$$

where *C*_0_ is the initial adsorbate concentration (mg/L), *C_e_* is equilibrium adsorbate concentration (mg/L), *V* is the volume of adsorbate solution (L), and *m* is mass of the adsorbent (g).

Pseudo-first order (PFO) (3), pseudo-second order (PSO) (4), and Elovich (5) models were applied to analyze adsorption kinetic data. Langmuir (6), Freundlich (7), Sips (8) and Redlich–Peterson (9) isotherm models were fitted to the adsorption equilibrium data. Non-linear model fitting was applied to calculate adsorption parameters.

(3) $$q_{t}=q_{e}\left(1-e^{-k_{1}t}\right)$$

(4) $$q_{t}=\frac{q_{e}{}^{2}k_{2}t}{1+k_{2}q_{e}t}$$

(5) $$q_{t}=\frac{1}{\beta }\ln\left(1+\alpha \beta t\right)$$

where: *q_e_* and *q_t_* are the amounts of adsorbate uptake per mass of adsorbent at equilibrium and at any time *t* (min), respectively; *k_1_* (1/min) and *k_2_* (g/(mg min)) are the rate constants of the PSO and PFO equation, respectively; *α* is the initial adsorption rate (mg/g min); and *β* is the desorption constant (g/mg).

(6) $$q_{e}=\frac{q_{m}K_{L}C_{e}}{1+K_{L}C_{e}}$$

(7) $$q_{e}=K_{F}C_{e}^{\;n}$$

(8) $$q_{e}=\frac{q_{m}{\left(K_{S}C_{e}\right)}^{m}}{1+{\left(K_{S}C_{e}\right)}^{m}}$$

(9) $$q_{e}=\frac{K_{RP}C_{e}}{1+\alpha_{RP}C_{e}{}^{g}}$$

where: *q_m_* (mg/g) is the maximum saturated monolayer adsorption capacity; *C_e_* (mg/L) is the adsorbate concentration at equilibrium; *q_e_* (mg/g) is the amount of adsorbate uptake at equilibrium; *K_L_* (L/mg) is Langmuir constant related to the affinity between adsorbent and adsorbate; *K_F_* is Freundlich constant [(mg/g)(L/mg)^n^], and *n* is the characteristic constant associated with the intensity of the sorption process; *K_S_* (L/mg) is the Sips constant, and *m* is a heterogeneity factor; *K_RP_* (L/g) and *α_RP_* (mg/L) are the Redlich–Peterson constants; and *g* is an exponent whose value must lie between 0 and 1.

## 3. Results and Discussion

### 3.1. Materials Characterization

**Figure 1:**
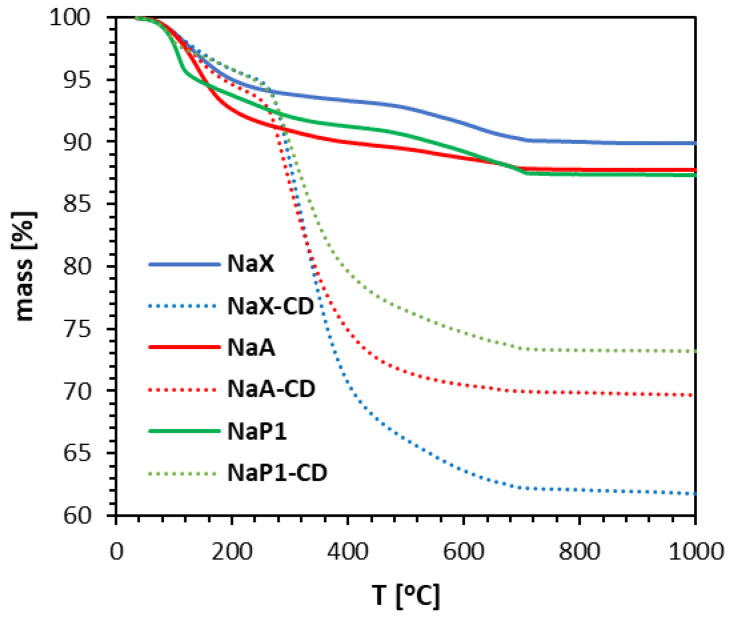
TG curves of zeolites NaX, NaA, and NaP1 before and after modification with β-CD.

TG curves of zeolites before and after their modifications depicted the percentage mass losses due to the constant heating of the samples up to 1000 °C (Figure 1). TG mass loss profiles for zeolites agreed well with the literature data [42,43]. The main mass loss occurred below 200 °C and is attributed to the release of adsorbed and zeolitic water [32,42,44]. Further weight loss can be attributed to dehydroxylation processes [45,46]. The total mass loss was 10.02%, 12.24% and 12.65% for NaX, NaA and NaP1, respectively. TG curves of zeolites after modification indicate mass losses associated with water release (up to about 180 °C), as well as significant mass loss attributed to dehydration and decomposition of organic matter (in the range about 250–400 °C) as reported for zeolites modified with chitosan [47,48] and cyclodextrin [36,39] The total mass loss was 38.2%, 30.28% and 26.75% for NaX, NaA and NaP1, respectively. In summary, the mass loss associated with the presence of the modifying compound can be reflected as 28.18% for NaX-CD, 18.04% for NaA-CD, and 14.10% for NaP1-CD, respectively, indicating the highest modification efficacy for the zeolite NaX, and the lowest for NaP1.

**Figure 2:**
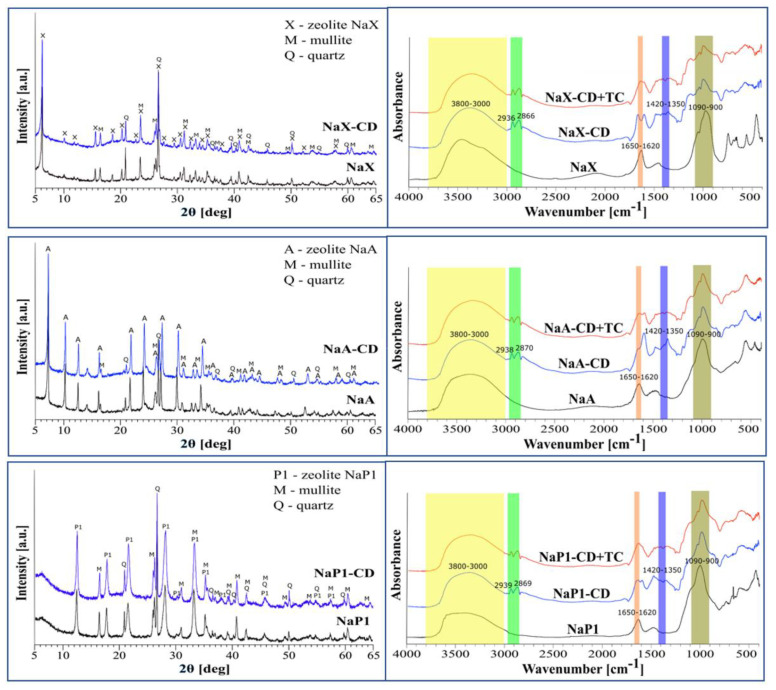
XRD patterns of NaX, NaA and NaP1 (**left column**) before and after modification with β-CD, and FTIR spectra of the studied organo-zeolites before and after TC adsorption (**right column**).

Mineral composition of the materials is presented in Figure 2 (left column). All the zeolites were composed of mineral phases attributed to NaX, NaA and NaP1 types, accompanied by some traces of mullite, quartz and amorphous glassy phase recognized by the characteristic rise in the background line in the angular range 15–35 °2θ.

The lower contribution of the amorphous phase is attributed to the NaX, suggesting the highest degree of crystallinity. The peaks characteristic for the zeolite phases can be recognized by the strongest reflections at the following d_hkl_ values: 14.45; 8.85; 3.34; 2.89, 2.79 Å for NaX; 12.23; 8.65; 3.70; 3.28; 2.98 Å for NaA; 3.17; 7.10; 4.10; 2.68; 5.02 Å for NaP1. It was in good agreement with the standards collected in the PDF2-2022 database. According to the database, the reference numbers are 00–038–0237 for NaX, 00–038–0241 for NaA, and 00–025–0778 for NaP1.

The reflections attributed to the zeolites are also present in the CD-modified materials, confirming that the modification process does not affect the zeolites’ mineral structure. Moreover, a characteristic ‘hump’ in the angular range from 15 to 25 °2θ can be identified, related to the occurrence of organic matter. A preliminary analysis of the diffractograms allows one to conclude that NaP1 was the least susceptible to modification with CD, which is in good agreement with TG results.

**Figure 3:**
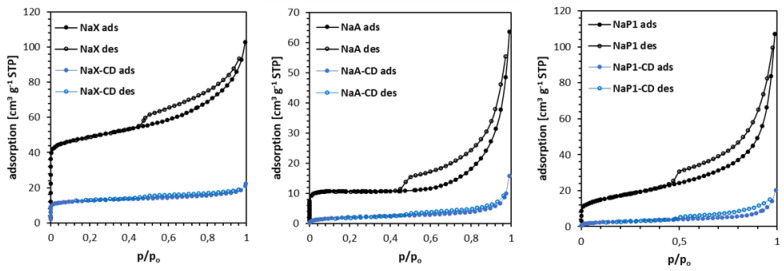
N_2_ adsorption–desorption isotherms of zeolites NaX (**left**), NaA (**middle**) and NaP1 (**right**) before and after modification with β-CD.

According to the IUPAC classification [49], the isotherms of the materials studied are a combination of types I and II (Figure 3). The hysteresis shapes can be attributed to the type H4, which is typical of aggregated zeolite crystals or mesoporous zeolites. All materials revealed narrow hysteresis loops, probably due to the tensile strength effect [50]. The rise in the nitrogen adsorption at low p/p_0_ values, related to micropore filling, is the highest for NaX, and higher than for NaA, thus revealing their microporous character. 

The modification with β-CD caused a dramatical decrease in the nitrogen adsorbed volume indicating the partial blocking of materials’ pores by β-CD. As a consequence, their volume decreased significantly. Table 3 shows textural parameters of the materials.

The most developed specific surface area and total volume of pores was noted for NaX. After the modification, a significant decrease in the *S_BET_* of the hybrid materials compared to the corresponding zeolites was observed, while this decrease was the largest for the zeolite NaP1 (by about 83%), than similar for NaA (76%), and for NaX (75%). The total volume of pores *V_tot_* decreased by about 78% for NaX, 75% for NaA, and 76% for NaP1. These textural changes suggest, that regardless of the structure type, *V_tot_* of zeolites after modification decreased similarly (in %). On the other hand, the specific surface of NaP1 decreased the most after the modification. That may suggest the modification mostly occurs on the external surface of materials.

**Figure 4:**
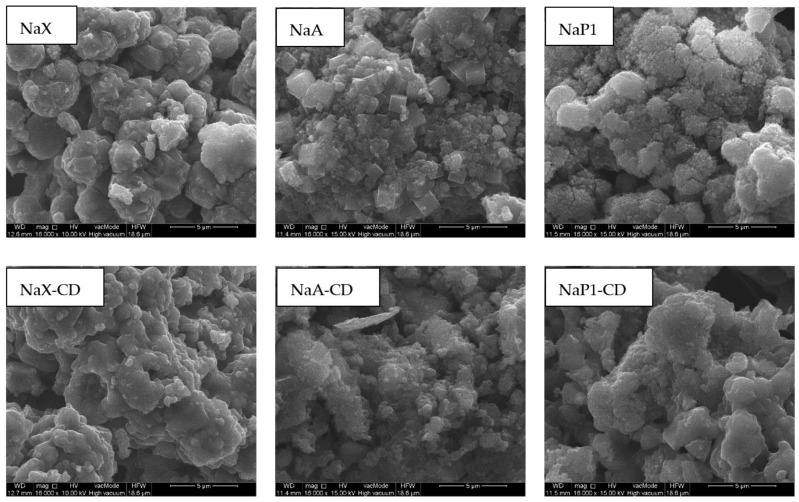
SEM images of NaX, NaA and NaP1 before and after modification with β-CD (magnification 16,000×).

Surface morphology of the zeolites before and after modification with β-CD was investigated via SEM (Figure 4). NaX occurs in a form of regular, isometric cubic crystals with the sizes of 2–6 µm, overlapping each other randomly. NaA has well-shaped cubic crystals with a size of 1–2 µm. Na-P1 occurs in the form of rosette-like adhesions composed of fine pillars. The sizes of aggregates usually exceed 10 µm in size. Significant changes in the morphological habit of the zeolites after the modification occurred. crystalic and well-shaped structures have been covered by the amorphous organic substance. The CD formed a specific layer that occluded the mineral material. This layer enveloped the crystalline forms and the spaces between them creating more rounded, softer shapes and globular structures. Similar forms were observed by us in previous work [36] as well as by Lv et al. [51] for pure CD, and for β-CD/Al(OH)_3_ composites [52].

**Figure 5:**
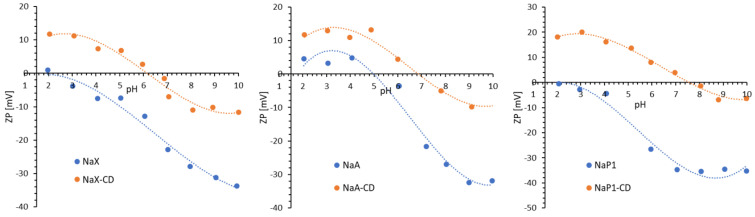
Zeta potential of zeolites NaX, NaA and NaP1 before and after modification with β-CD.

Electrokinetic properties of solid particles dispersed in aqueous media provides information on the solid surface chemical nature. The charge density of the diffusion layer within the electric double layer (EDL) formed on the surface of a solid particle depends on the value of the zeta potential (ζ). The charge of the diffusion layer increases when a displacement of counter ions perpendicular to the surface takes place, which causes an increase in the ζ-potential [53]. In the case of the zeolites studied, the attachment of β-CD molecules to their surface (via the linker GLYMO), which requires the formation of two bonds, probably results in the removal of divalent cations present in the zeolite structure. This displacement of counter ions in the direction perpendicular to the zeolite surface is therefore the result of the active sites blocking on the solid surface by the adsorbed modifier molecules. Figure 5 shows ζ-potential for the zeolites before and after modification. An increase in the ζ-potential was observed over the entire pH range after CD modification, as well as a significant shift in the position of their isoelectric points (IEP) towards higher pH values—the highest for the zeolite NaP1 (pH_IEP_ increased from a value of about 2 for the initial NaP1 to a value of about 8 for the modified NaP1-CD). Similar behavior was observed by other authors who studied the interactions of dextrins with the surface of various solids [54] and sepiolite treated with organosilanes [55].

The FTIR spectra of the zeolite materials NaX, NaA and NaP1 (Figure 2, right column) are characterized by the presence of bands typical for zeolite structures of the faujasite (FAU), zeolite A (LTA) and gismondite (GIS) groups, respectively [42,56,57]. The spectra of the hybrid materials (NaX-CD, NaA-CD and NaP1-CD) confirm the effectiveness of the modifications using β-CD, revealing the presence of bands characteristic of zeolites and β-CD. The bands with a maximum in the range of 3800–3000 cm^−1^ are attributed to the stretching vibrations of the O-H groups present on the materials’ surface. In the case of the hybrid materials, two characteristic bands originating from asymmetric vibrations of -CH_2_- groups and symmetric stretching vibrations of -CH_3_ and -CH_2_- groups can be distinguished, located at 2936 and 2866 cm^−1^ for NaX-CD, 2938 and 2870 cm^−1^ for NaA-CD, and 2939 and 2869 cm^−1^ for NaP1-CD. These bands are indicative of the presence of the hydrocarbon chain and methyl group of the linker molecule, and may also be related to the vibration of the hydrocarbon groups present in β-CD. Bands at wavenumbers in the range of 1650–1620 cm^−1^ are associated with bending vibrations of the O-H groups of the so-called zeolite water. The band at about 1450 cm^−1^, present for all zeolite materials, indicates the presence of carbonates. On the spectra of the hybrid materials, significant changes are observed in the wavenumber range of 1420–1350 cm^−1^. In this region, vibrations originating from the glucose units that build the CD cup are present, and the visible changes indicate the effectiveness of the modification performed. Furthermore, the presence of a band at about 1200 cm^−1^ on the spectra of the hybrid materials indicates the chemical bonding of the linker molecule (GLYMO) to the surface of the zeolite materials. For all zeolite materials, we observe the presence of a strong band in the range of 1090–900 cm^−1^, which is attributed to the symmetric and asymmetric stretching vibrations of the Si-O(Al) and Si-O(Si) bridge bonds. Due to the modification process, the intensity of this band decrease and its shape change which is related to the presence of glucosidic -O-C-C stretching vibrations of β-CD units. In zeolite-CD-TC complex, slight changes in the bond shapes at the wavelength range 1200–1700 cm^−1^ can be noticed that may indicate the occurrence of new oxygen-containing groups and/or changes in the surface functional groups of the material after TC adsorption. These observation is consistent with the spectra obtained for core–shell TiO2@C ultralong nanotubes TC adsorption [58].

**Figure 6:**
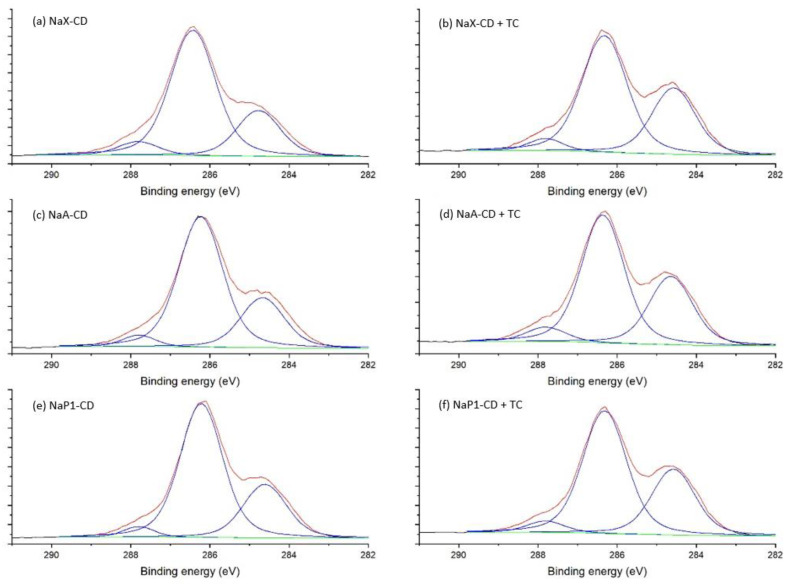
High-resolution C1s spectra of NaX-CD (**a**), NaX-CD after TC adsorption (**b**), NaA-CD (**c**), NaA-CD after TC adsorption (**d**), NaP1-CD (**e**), NaP1-CD after TC adsorption (red—experimental curve, blue—fit peak, green—background) (**f**).

XPS was employed to explore the chemical composition of the materials’ surface and binding energies of adsorbents before and after adsorption of TC (Figure 6). As can be seen in Table 4, the surface of fly ash zeolites is mainly composed of oxygen (58.8–62.0%), silicon (14.4–19.0%) and aluminum (5.6–9.4%) atoms. The basic chemical composition is complemented by mono- and divalent metal atoms such as sodium (3.6–6.7%), potassium (0.5–0.8%, magnesium (1.6–6.3%) and calcium (2.5–3.5%). Carbon (0.8–1.3%) and iron (0.3–1.3%) were found in much smaller amounts on the surface of zeolites. After modification with β-CD, the chemical composition of the materials’ surface changed significantly. The vast majority of atoms were oxygen (37.6–41.0%) and carbon (48.2–53.7%), associated with molecules of the organic modifier. Other elements, such as silicon and aluminum, were present in small amounts of 6.4–7.1%, which is due to the deposition of the modifier molecules on the surface of the materials. There was also a significant decrease in mono- and divalent metal atoms which play the role of exchangeable cations.

The small amount of nitrogen in zeolites after β-CD modification was originating from the DMF used during the synthesis while N1s peak was not observed in bare zeolites. The N1s spectra of the NaX-CD, NaA-CD and NaP1-CD showed two peaks at around 399.4 eV and 402.5 eV, which were assigned to the amide and protonated N, respectively (data not shown). The proportion of N1s slightly increased after TC adsorption and the peaks were found at similar binding energies (BEs) with no significant changes in their proportions. The peak at 399.4 eV included tetracycline’s amine and amide nitrogen.

**Figure 7:**
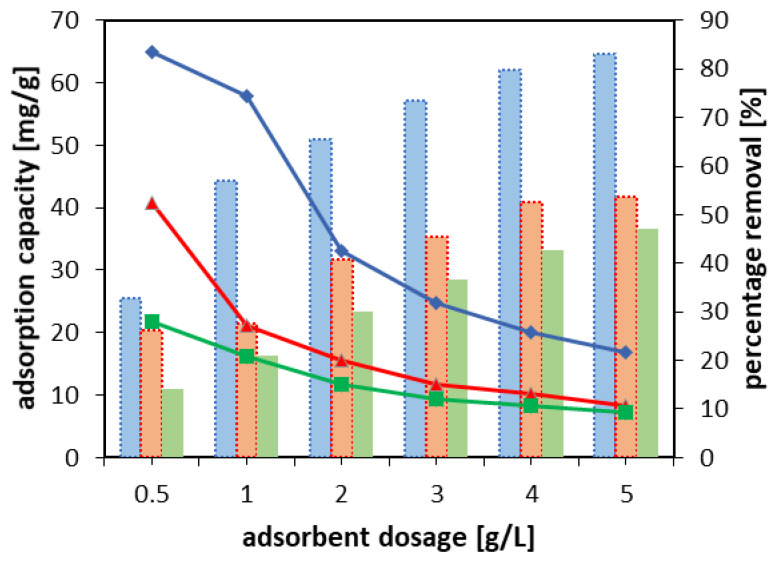
The effect of the adsorbent dosage, V = 25 mL, C_0_ = 100 mg/L; lines, attributed to adsorption capacity (mg/g), NaX-CD (blue); NaA-CD (red); NaP1-CD (green); bars, to percentage removal (%), NaX-CD (blue); NaA-CD (orange); NaP1-CD (green).

The C1s spectra of the hybrid materials NaX-CD, NaA-CD and NaP1-CD were fitted with three peak components (Figure 7). The first component with (BEs) at 284.6–284.8 eV belongs to the C–C/C–H bonds. The middle component centered at 286.2–286.4 eV was associated with C–N (from DMF) and C–O bonds. The third component at 287.8 eV was associated with O–C–O bonds of the β-CD structure [59] and N–C=O of the DMF. After TC adsorption, the BEs of these components were similar (±0.1 eV), while the proportion of the first component clearly increased (from 24–28% to 33–34%). This was due to introducing a higher proportion of C–C/C–H bonds and, additionally, aromatic carbon by the TC.

### 3.2. Adsorption Study

#### 3.2.1. Effect of Adsorbent Dose

The experiments of adsorbent dosage were performed with the initial TC concentration of 100 mg/L and solution volume of 25 mL. The results revealed that the highest adsorption capacity was obtained using the dosage of 0.5 g/L; thus, it was chosen for the further adsorption studies (Figure 7). On the other hand, the greater was the dosage, the higher was the percentage removal of TC obtained, which reached 83% for NaX-CD, 54% for NaA-CD, and 47% for NaP1-CD at the adsorbent dosage of 5 g/L. This was due to the increasing number of binding sites when more sorbent was available in the system [60].

#### 3.2.2. Effect of Contact Time and Adsorption Kinetic Study

**Figure 8:**
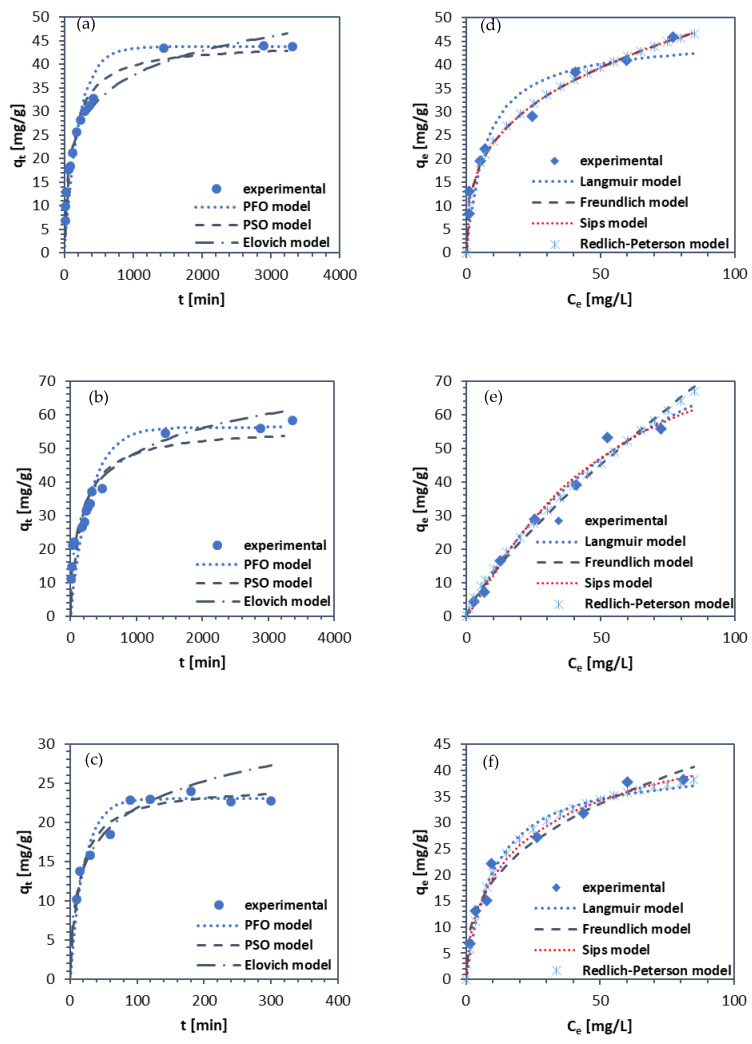
Adsorption of TC with experimental points and calculated fitted models: kinetic plots for (**a**) NaX-CD, (**b**) NaA-CD, (**c**) NaP1-CD; isotherm plots for (**d**) NaX-CD, (**e**) NaA-CD, (**f**) NaP1-CD.

The adsorption rate was the highest within circa first 200 min. It was also an optimal time for NaP1-CD to achieve the adsorption equilibrium (Figure 8). Table 5 summarizes the kinetic parameters obtained for TC adsorption on zeolites modified with β-CD. According to the rate constants determined from PFO and PSO, TC adsorption was the fastest for NaP1-CD by more than 1 order of magnitude than for NaX-CD and NaA-CD. Adsorption rate for NaX-CD was slightly higher than for NaA-CD. The calculated values *q_c_* was in good agreement with the experimental *q_e_*. The best fitting was achieved for the Elovich model, followed by the PSO model. The Elovich model is useful in describing chemisorption (with new chemical bonds formation [61]) on heterogeneous adsorbents. Good fitting to PSO was also obtained for tetracycline adsorption onto graphene oxide [62], hierarchical porous MIL-53(Cr) (in this case, the Elovich model also provided good fitting) [63] and metal- and clay-embedded cross-linked chitosan [3].

Most authors claimed PSO model indicates TC adsorption via electron coalescence or electron exchange. However, as reported by Tran et al. [64] and rightly pointed out by Gogoi et al. [60] adsorption mechanisms cannot be directly determined based only on PFO and PSO models, but more extensive studies should be performed. Nevertheless, taking into account the good fitting to the PSO and Elovich models, as well as the chemical structure of TC and the adsorbents’ surface, chemical adsorption including, e.g., hydrogen bonding is highly probable.

#### 3.2.3. Effect of Initial Concentration and Adsorption Isotherms

Adsorption capacities (mg/g) at different initial concentrations (10–100 mg/L) of TC of investigated sorbents and Langmuir, Freundlich, Sips, and Redlich–Peterson isotherm model fitting are shown in Figure 8d–f. The maximum *q_e_* from the experiment was around 48 mg/g for NaX-CD, 60 mg/g for NaA-CD, and 38 mg/g for NaP1-CD. That revealed the adsorption efficacy towards TC follows the order NaA-CD > NaX-CD > NaP1-CD. The lowest adsorption capacity for NaP1-CD can be attributed to the lowest modification efficacy as it is revealed by the TG curves (Figure 1). Despite significant differences in textural properties (Table 3), NaX and NaA zeolites showed very high similarities in the amount of attached CDs, as best indicated by XPS results. Although TG results indicate that NaX-CD showed the highest weight loss indicating the best modification efficiency (Figure 1), it is not fully clear how much of this loss was actually related to the CD and how much to the attached amount of silane linker (GLYMO). Thus, it can be speculated that the NaX-CD and NaA-CD adsorbents showed very similar outer surface with respect to the presence of the CD modifier. Undeniably, TC adsorption efficiency is closely related to the number of active sites on the adsorbent, in this case most likely CD cups. The more of them, the higher the sorption capacity is. It can be observed that despite the similar quantitative and qualitative chemical surface of NaX-CD and NaA-CD, they show slight differences in sorption capacity for the same substance. Therefore, other factors which may affect the adsorption should be taken into account and need to be further investigated.

Table 6 presents adsorption isotherm parameters. Among used models, it transpired that the Sips isotherm model was the most suitable to describe adsorption process on each material (the highest R^2^). The Sips model combines the principles of both Langmuir and Freundlich. Additionally, the Redlich–Peterson model (R-P) revealed good fitting. In the R-P, when g = 1, the equation is equal to the Langmuir model, and when g = 0, the adsorption follows Henry’s law. In the case of NaP1-CD, g was close to 1. In the case of NaA-CD, Langmuir model fitted also well with the R^2^ equal 0.986. The obtained parameters may indicate the adsorption onto the uniform and equivalent sites with a binding power (i.e., adsorptive strength) between the adsorbents and adsorbate (which can be a dominant mechanism) as well as heterogeneous adsorption (which can occur to a lesser degree). Foroutan et al. [65] reported good fitting of the R-P model for TC adsorption onto carbon nanotubes/CD magnetic composites. One of the possible bindings between CD and TC is hydrogen bonding, including hydroxyl groups of CD’s cups and the -NH2 group present in TC.

Table 7 shows adsorption capacities obtained in the literature for removal of TC from water by using various adsorbent materials. These reports revealed that zeolites from fly ash modified by CDs can be placed among promising, sustainable materials for TC adsorption. Moreover, the possibility of its fabrication from waste materials, and the ease of regeneration (confirmed previously in [36]) are advantages that make these hybrid organo-mineral adsorbents a viable alternative to activated carbons.

## 4. Conclusions

In this study, three different zeolite types NaX, NaA, and NaP1 were synthesized from fly ash and modified with CD with a view to tetracycline adsorption from aqueous solution. It was shown that the zeolite structure of NaP1 was less susceptible to the modification process than NaX and NaA, and this was due to different structure and lower binding of modifier, as indicated by TG results. The textural properties of the starting materials changed under the modification, which was due to the coating of the surface with modifier molecules that blocked the pores. It was noticed that despite different *S_BET_*, NaX and NaA revealed similar susceptibility to modification with CD and similar adsorption efficiency towards tetracycline. Tetracycline adsorption capacity followed the order NaA-CD > NaX-CD > NaP1-CD. On the other hand, the NaP1-CD material showed a much higher removal rate, as demonstrated by the experimental results and the kinetic models used. The adsorption process can be well described by the Sips and Redlich–Peterson isotherm models, which both combine principles of the Langmuir and Freundlich models. It is very likely that adsorption mechanisms are complex, with dominating adsorption onto energetically homogeneous active sites which are CD cups, with the possibility of strong interactions between CD’s hydroxyl groups and amine group present in tetracycline. Zeolites from fly ash modified with CD exhibit good potential as alternative adsorbents in the water treatment technologies aimed at pharmaceuticals removal from the aquatic environment.

## Figures and Tables

**Table 1:** Chemical composition of fly ash.

Compound	Na_2_O	MgO	Al_2_O_3_	SiO_2_	P_2_O_5_	SO_3_	K_2_O	CaO	TiO_2_	Fe_2_O_3_	LOI
Content [%]	nd	0.95	25.80	51.64	1.76	0.66	3.02	3.05	1.85	7.22	3.29

**Table 2:** Conditions applied for the zeolites synthesis.

Material	Fly Ash [kg]	NaOH Concentration [M]	NaOH Volume [dm^3^]	Time [h]	Temperature [°C]	Aluminum Source [kg]
NaX	20	3	80	48	70	–
NaA	20	3	80	10	80	1.44
NaP1	20	3	90	48	90	–

**Table 3:** Textural parameters of NaX, NaA and NaP1 before and after modification with β-CD.

Material	NaX	NaX-CD	NaA	NaA-CD	NaP1	NaP1-CD
S_BET_ [m^2^/g]	164	41	33	8	59	10
V_tot_ [cm^3^/g]	15.90 × 10^−2^	3.48 × 10^−2^	9.85 × 10^−2^	2.44 × 10^−2^	12.97 × 10^−2^	3.13 × 10^−2^

S_BET_, specific surface area; V_tot_*,* total pore volume calculated at p/p_0_ = 0.98.

**Table 4:** Elemental composition (atomic concentration %) of the surface of the bare zeolites and zeolites modified with β-CD before and after TC adsorption.

Element	NaX	NaA	NaP1	NaX-CD	NaA-CD	NaP1-CD	NaX-CD+TC	NaA-CD+TC	NaP1-CD+TC
O	60.3	58.8	62.1	41.0	39.3	37.6	34.6	34.3	33.5
Mg	6.3	4.7	1.6	0.3	-	-	0.4	0.2	0.9
Si	17.7	14.4	19.0	7.3	6.4	7.1	5.1	5.4	5.9
Al	6.1	9.4	5.6	1.1	0.8	0.3	0.7	0.6	-
Na	3.6	6.7	5.9	0.3	0.4	-	0.6	1.5	-
Ca	3.4	2.5	3.5	0.5	-	-	0.6	-	0.6
K	0.6	0.5	0.8	-	-	-	-	-	-
C	1.3	1.3	0.8	48.2	51.8	53.7	53.8	53.8	55.2
Fe	0.5	1.3	0.3	-	-	-	-	-	-
Zn	0.4	0.4	-	-	-	-	-	-	-
Ti	-	-	0.4	-	-	-	-	-	-
N	-	-	-	1.3	1.4	1.3	2.6	1.5	2.5
Cl	-	-	-	-	-	-	1.7	2.0	1.5
F	-	-	-	-	-	-	-	0.7	-

**Table 5:** Kinetics parameters for the adsorption of tetracycline on NaX-CD, NaA-CD and NaP1-CD.

Kinetic Model	NaX-CD	NaA-CD	NaP1-CD
Pseudo-first order			
q_c_ [mg/g]	43.71	56.22	23.00
k_1_ [min^–1^]	4.80 × 10^−3^	3.41 × 10^−3^	4.76 × 10^−2^
R^2^	0.886	0.760	0.897
Pseudo-second order			
q_c_ [mg/g]	44.43	56.22	24.68
k_2_ [g/mg min]	1.873 × 10^−4^	1.116 × 10^−4^	2.881 × 10^−3^
R^2^	0.963	0.787	0.945
Elovich			
α (mg/g min)	1.119	1.083	3.983
β (g/mg)	0.133	0.095	0.201
R^2^	0.977	0.910	0.971

where *q_c_* is the amount of adsorbate uptake per mass of adsorbent at equilibrium, calculated from PFO and PSO models; other parameters are explained in Section 2. Materials and Methods.

**Table 6:** Isotherm parameters for the adsorption of tetracycline on NaX-CD, NaA-CD and NaP1-CD.

**Isotherm Model**	**NaX-CD**	**NaA-CD**	**NaP1-CD**
Langmuir			
q_m_ [mg/g]	45.86	124.52	41.36
K_L_ [L/mg]	0.141	0.012	0.100
R^2^	0.911	0.986	0.961
Freundlich			
K_F_ [(mg/g)(L/mg)^1/n^]	11.09	2.13	8.25
n	0.324	0.781	0.359
R^2^	0.952	0.963	0.955
Sips			
q_m_ [mg/g]	6879.50	94.41	63.03
K_S_	0.001614	0.010349	0.11319
m	0.326	1.169	0.600
R^2^	0.986	0.987	0.977
Redlich–Peterson			
K_RP_	115.46	45.87	4.93
α_RP_	9.72	16.56	0.18
g	0.692	0.279	0.907t
R^2^	0.987	0.976	0.970

*q_e_*_,_ experimental adsorption capacity in equilibrium (mg/g); R_2_ is the non-linear regression coefficient; other parameters are explained in the Section 2*. Materials and methods.*

**Table 7:** Comparison of the adsorption capacity of TC on different adsorbents.

Material	Adsorption Capacity [mg/g]	Reference
Na-montmorillonite	49.3	[66]
Kaolinite	4.0	[67]
Biochars	6.5–14.2	[68]
poly(vinylidene fluoride)/polyaniline-montmorillonite mixed matrix membranes	1.4; 3.9; 25.1; 51.0	[69]
Rice husk ash	4.0	[70]
Porous carbon from waste hydrochar	25.0	[71]
Chitosan/Olive pomace film; olive pomace	1.60; 16.0	[72]
ChitosanZirconium-embedded chitosanZirconium- and perlite-embedded chitosan	5.015.045.0	[3]
Carbon nanotubes/β-cyclodextrin/MnFe2O4	82.7–89.5	[65]
NaX-CD; NaA-CD; NaP1-CD	48.0; 60.0; 38.0	This study

## Data Availability

Not applicable.

## Supplementary Materials

The following supporting information can be downloaded at: https://www.mdpi.com/article/10.3390/ma15186317/s1, Table S1.
